# Effect of a 12-Week Multicomponent Program on Mental Disorders as Well as Biochemical and Immunological Parameters in Teachers with Overweight

**DOI:** 10.3390/biomedicines13102354

**Published:** 2025-09-25

**Authors:** Ricelli Endrigo Ruppel da Rocha, Sergio Fallone de Andrade, Adriano Alberti, Roberto Moraes Cruz, Fatima Noely da Silva, Diego André Bridi, Jaqueline Tellis de Souza, Fábio Pitanga, Rafael Bianchi, Débora Fernandes Pinheiro, Bruna Becker da Silva, Clayton Luiz Zanela

**Affiliations:** 1 Faculty Member of the Graduate Program in Basic Education and Development and Society, Alto Vale do Rio do Peixe University (UNIARP), Caçador 89500-064, Brazil; 2 Laboratory of Systems Integration Pharmacology, Faculty of Pharmacy, Lisbon University, 1600-083 Lisbon, Portugal; sfallone@ff.ulisboa.pt; 3 Graduate Program in Environment and Health, University of Planalto Catarinense—UNIPLAC, Lages 88509-900, Brazil; adrianoalberti90@hotmail.com; 4 Faculty Member of the Graduate Program in Psychology, Federal University of Santa Catarina (UFSC), Florianópolis 88040-900, Brazil; robertocruzdr@gmail.com; 5 Master’s and Doctoral Program in Basic Education and Master’s and Doctoral Program in Development and Society, Alto Vale do Rio do Peixe University (UNIARP), Caçador 89500-000, Brazil; fatima@uniarp.edu.br (F.N.d.S.); diego@uniarp.edu.br (D.A.B.); jaqueline.souza@uniarp.edu.br (J.T.d.S.); fabio.pitanga@uniarp.edu.br (F.P.); rafaelbianchi17@gmail.com (R.B.); uniarp@uniarp.edu.br (D.F.P.); clayton@uniarp.edu.br (C.L.Z.); 6 Department of Biological and Health Sciences Program in Health Sciences, University of Southern Santa Catarina (UNISUL), Palhoça 88132-260, Brazil; brunabecker__@hotmail.com

**Keywords:** education, exercise, therapy, nutrition

## Abstract

**Background:** To reduce work-related illnesses among teachers, various types of programs were implemented. **Objectives:** The aim of this study was to evaluate the effects of a 12-week multicomponent program on mental disorders, biochemical parameters, and immunological markers in female teachers with overweight. **Methods:** A total of 33 women who were basic education teachers with a body mass index (BMI) ≥ 25 kg/m^2^ participated in this study. Participants were randomly assigned to either a control group (*n* = 16), which did not participate in the program, or an intervention group (*n* = 17), which underwent the multicomponent intervention. The program included physical exercise (three sessions per week), cognitive–behavioral therapy delivered monthly across three modules, and nutritional education consisting of both general and specific guidance. Assessments were conducted at baseline and after 12 weeks and included measurements of symptoms of depression, anxiety, and stress; fasting glucose; total cholesterol; LDL cholesterol; HDL cholesterol; VLDL cholesterol; triglycerides; and concentrations of immunoglobulins IgA, IgG, and IgM. **Results:** After 12 weeks, the intervention group showed a significant reduction in symptoms of depression, anxiety, and stress (*p* < 0.05), as well as in fasting glucose, triglycerides, and VLDL cholesterol levels compared to the control group (*p* < 0.05). No significant changes were observed in the levels of immunoglobulins IgA, IgG, or IgM (*p* > 0.05). **Conclusions:** The multicomponent program improved mental health and reduced the risk of developing metabolic and cardiovascular diseases in female teachers with overweight.

## 1. Introduction

Teaching is widely recognized as one of the most demanding professions and carries a high risk for the development of respiratory and musculoskeletal diseases, voice disorders, and mental health conditions—particularly depression, anxiety, and stress [1,2]. Moreover, many teachers lead sedentary lifestyles, have low physical fitness levels, and are overweight, which significantly increases their risk for developing other health conditions, such as cardiovascular diseases [3,4,5]. In Brazil, most teachers work in basic education, with 2.2 million professionals making up one of the largest employment categories and playing a fundamental role in the country’s development and social stability [6]. Despite the importance of teachers to society and future generations, they are among the workers with the highest rates of sick leave due to health-related issues [7].

In a study involving basic education teachers from the state of São Paulo, 56% reported missing at least one day of work due to health problems [8]. The Educatel population-based study, which assessed a sample of 5116 basic education teachers from 24 Brazilian state capitals between 2015 and 2016, found that 54.0% of female teachers and 44.0% of male teachers were absent from work for at least one day due to illness [9]. Teachers’ absence from work impacts management and school routine, increases the costs of recruiting new teachers, and harms student performance and learning. The effects are loss of productivity, limitations and suffering, causing a reduction in well-being at work [8].

Given this context, an increasing number of interventions have been implemented in teachers’ work environments to reduce symptoms of stress, anxiety, depression, and burnout, as well as to improve overall and occupational well-being. Mindfulness programs, practices based on positive psychology, interventions aimed at mitigating negative emotions, and the promotion of physical activity and yoga are among the most commonly applied strategies [10,11,12,13,14,15]. Although these programs have generally shown benefits for improving teachers’ physical and mental well-being, in most of the studies, only one or two types of interventions were implemented. Therefore, interventions involving more than two components have not been thoroughly evaluated, highlighting the need for further research in this area. Moreover, we found no studies investigating the effects of such programs on biochemical and immunological parameters related to chronic diseases in basic education teachers.

Therefore, this study aimed to assess the effects of a 12-week multicomponent program comprising physical exercise, cognitive behavioral therapy, and nutritional education on mental disorders, biochemical parameters, and immune function in basic education teachers with overweight. We hypothesize that the multicomponent program will reduce risk factors for the development of mental and cardiometabolic diseases in basic education teachers.

## 2. Materials and Methods

### 2.1. Participants

This study included teachers working in basic education at public municipal schools in the city of Caçador, located in the Midwest region of the state of Santa Catarina, Brazil. The following inclusion criteria were applied: (a) female teachers; (b) actively teaching at the early childhood, elementary, or high school levels; (c) a minimum of two years of teaching experience; (d) classified as overweight (BMI ≥ 25 kg/m^2^) [16]; (e) available to attend the program three times a week in the afternoons, after 5:00 p.m.; (f) had not engaged in physical exercise for at least one year and; (g) did not have any physical or mental conditions that would prevent them from taking part in this study.

To carry out this research, authorization was first requested from the municipal Secretary of Education and approved by the Research Ethics Committee of Alto Vale do Rio Peixe University for studies involving humans. Subsequently, meetings were scheduled with school principals to arrange times to present this study’s objectives and procedures to the teachers. After this initial phase, the researchers returned to the schools to conduct anthropometric assessments of body weight and height to determine overweight status among the teachers.

Body weight was measured using a digital scale (Filizola^®^, São Paulo, SP, Brazil), with a capacity of up to 250 kg and an accuracy of 100 g, with the teachers barefoot, wearing minimal clothing, and without any objects in their pockets, hands, or on their heads. Height was measured using a portable stadiometer (Avanutri^®^, Três Rios, RJ, Brazil) millimeter-marked, with an accuracy of up to 1 mm. The teachers stood upright, barefoot, with their upper limbs hanging along the body.

In total, 90 female teachers were assessed, and, of these, 33 met the inclusion criteria and agreed to participate in this study. All signed the free and informed consent form (FICF), and this study was approved by the research ethics committee of the university referred to as Universidade Alto Vale do Rio do Peixe (UNIARP), under protocol code 49666115.3.0000.5593.

### 2.2. Procedures

**Figure 1:**
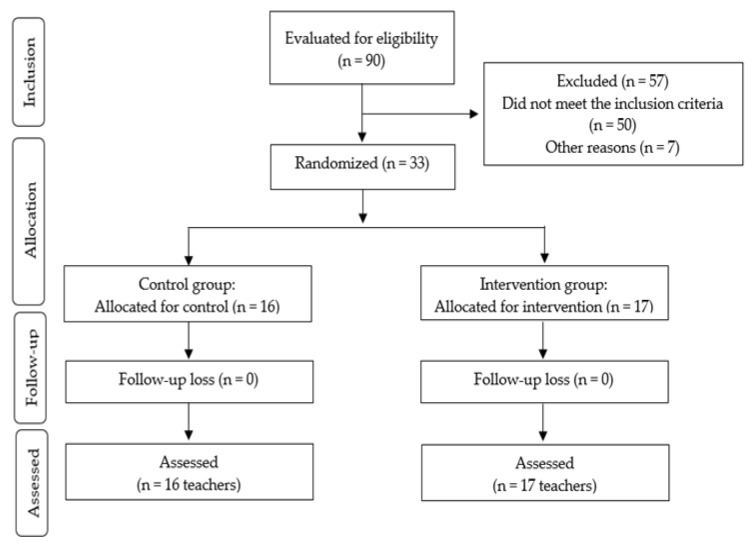
Flowchart illustrating the allocation of participants into the intervention and control groups.

Initially, participants were randomly assigned to one of two groups: (1) an intervention group (*n* = 17), which participated in a 12-week multicomponent program consisting of physical exercise, cognitive–behavioral therapy, and nutritional education; and (2) a control group (*n* = 16), which did not participate in the program and was instructed to continue their usual daily activities throughout the study period. Figure 1 presents the flowchart of participant allocation across the study groups.

### 2.3. Multicomponent Program

The multicomponent program consisted of physical exercises, cognitive–behavioral therapy, and nutritional education. For the physical exercises, the circuit training method [17] was used, with a duration of 60 min, divided into warm-up (10 min), main session (40 min), and cool-down (10 min), three times a week (Mondays, Wednesdays, and Fridays) between 5:00 p.m. and 6:00 p.m., for 12 weeks. All of them signed an attendance list containing the day and month before starting each physical exercise session with the aim of controlling the frequency of weekly participation in the program.

During the warm-up, the teachers performed joint mobility exercises for the trunk, spine, shoulders, hips, knees, and ankles. The main session was divided into 10 stations of resistance exercises for the major muscle groups, one station for walking/running on a treadmill, and another station on a stationary bike, totaling 12 exercises. The teachers performed 30 s of exercise at each station with 30 s of recovery (transitioning from one exercise to another), completing three sets of 10 to 15 repetitions per station. The resistance exercise load was adjusted for each participant using the session Rating of Perceived Exertion (s-RPE) [18]. To familiarize the teachers with the s-RPE method, the teachers performed 3 familiarization sessions with the s-RPE method, interspaced by 72 h. Sessions were composed of progressive increases in training load (weight), allowing subjects to experience different loads (i.e., light= 3 × 15 RM; moderate= 3 × 12 RM and; heavy= 3 × 10 RM) that would be used in the present study. Two minutes of rest were allowed between sets.

The training load was adjusted according to each subject’s s-RPE. Thirty minutes after the end of each training session, subjects were asked, “How was your workout?” Subjects answered the question according to the CR-10 scale [18]. They were instructed to choose a descriptor and its respective number from 0 to 10 (0 = rest; 1 = very easy; 2 = easy; 3 = moderate; 4 = somewhat hard; 5–6 = hard; 7–9 = very hard; 10 = maximum).

To adjust the progression of training load, all subjects initially started with the same exercise protocol (i.e., weeks 1–4; 3 × 15 RM). For subsequent training sessions, the exercise workload was individually adjusted based on the s-RPE response from the previous session. If the s-RPE values were between 5 and 6 (i.e., hard), the workload was maintained for the next session.

Between weeks 5 and 8, the load was increased and the number of repetitions decreased (3 × 12 RM), with s-RPE values ranging between ≥6 and ≤7 (hard). In the final weeks (weeks 9–12), the load was further increased and repetitions were reduced (3 × 10 RM), with s-RPE values ranging between ≥7 and ≤8 (hard to very hard).

During the cool-down, the teachers performed stretching exercises for the main joints, with each exercise lasting 20 to 30 s. The entire exercise program was conducted at the Health and Well-Being Innovation Laboratory (LABISA) of the Alto Vale do Rio do Peixe University (UNIARP). Table 1 presents the exercise program.

The cognitive–behavioral therapy interventions were developed according to the proposal by Fava et al. [19], with a total workload of 3 h, divided into 3 modules, conducted once a month. All activities were carried out by a psychology professional in the lecture room of the Teaching Health and Well-Being Innovation Laboratory at UNIARP. To control the frequency of participation in therapies at this stage, all participants signed an attendance list at the beginning and end of the intervention that contained the title of the activity, the date and the day it was carried out. Table 2 presents the activities.

The educational nutritional intervention for the teachers consisted of two parts: the first was a general guidance activity, and the second was specific guidance. All activities were conducted by a professional nutritionist at the Nutrition Clinic of Alto Vale do Rio do Peixe University (UNIARP). The teaching methodology included interactive lessons with visual resources, a set of transparencies, and workshops focused on awareness and food tasting.

The total workload was 3 h, divided into 3 classes, held once a month. The curriculum was defined by the nutritionist, and the topics covered during the educational nutritional intervention included the importance of nutrition for teachers’ health; food groups and their functions in the body; specific nutritional needs and recommendations for the teacher population; and self-care in nutrition.

To control the frequency of participation in activities at this stage, all participants signed an attendance list at the beginning and end of the intervention that contained the title of the activity, the date and the day it was carried out.

### 2.4. Study Design

All assessments of biochemical and immunological parameters and mental disorders were carried out (a) at the beginning of the multicomponent program (first week of September) and (b) after 12 weeks of multicomponent program (last week of November). The assessments of biochemical and immunological parameters (day 1) were carried out in the clinical analysis laboratory Madalozo Camatti Ltd.a (Caçador, Brazil), while the assessment of mental disorders (day 2) was carried out at the Laboratory of Innovation in Health and Teacher Well-Being (LABISA) at the Alto Vale do Rio do Peixe University—UNIARP.

### 2.5. Biochemical Assessment

Fasting glucose and lipid profile (total cholesterol, LDL-c, HDL-c, VLDL-c, and triglycerides) were measured using enzymatic colorimetric methods, whereas immunoglobulins (IgA, IgG, IgM) were determined by immunoturbidimetric assay, following standard laboratory procedures.

Venous blood sampling

To obtain the blood samples, the teachers fasted for 8 to 12 h and were instructed to avoid alcoholic beverages, excessive fat intake, and physical activity for 48 h prior to the tests. The blood collection was carried out in the morning (7:00 to 9:00 a.m.). Blood without anticoagulants was collected, and the serum was centrifuged soon after so as to perform the tests. Plasma was quickly frozen and stored at −70 °C. Whole-blood count and some plasma and clinical parameters were measured in samples according to the laboratory standard operating procedures.

Blood biochemical evaluation

The blood biochemical evaluation verified the levels of fasting glucose, total cholesterol, low-density lipoprotein cholesterol (LDL-c), high-density lipoprotein cholesterol (HDL-c), very-low-density lipoprotein cholesterol (VLDL-c), and triglycerides of the teachers. The enzymatic colorimetric method was applied by using 5 mL of serum after centrifugation at 3000 rpm for five minutes. The dosages were performed by applying the automated method in the Labmax Plenno appliance and are expressed in milligrams per deciliter (mg/dL).

The blood biochemical evaluation of immunoglobulin A (IgA), G (IgG) and M (IgM) was measured in plasma samples by immunoturbidometric assay (Pentra 400 autoanalyser, Horiba, France) according to the recommendations of the manufacturer. The immunoglobulins are expressed in milligrams per deciliter (mg/dL).

### 2.6. Mental Disorder Assessments

To assess mental disorders, the version of the Depression, Anxiety, and Stress Scale (DASS-21) adapted for Brazilian adults proposed by Vignola et al. [20] was used. In the DASS-21, participants indicate the degree to which they experienced each of the symptoms described in the items during the past week (previous week) using a 4-point Likert scale ranging from 0 (does not apply to me) to 3 (applies very much to me or most of the time). Scores for depression, anxiety, and stress are determined by summing the scores of the 21 items. The resulting scores classify the levels of depression, anxiety, and stress into different severity categories: normal, mild, moderate, severe, and extremely severe. The entire administration and analysis of the scale were conducted by a professional psychologist.

### 2.7. Statistical Analyses

The data descriptive analysis was carried out, and results are shown as mean, standard deviation (SD). In order to determine the parametric or non-parametric statistics, data normality was verified with Shapiro–Wilk test and Levene’s test to assess the homogeneity of the variables. Student’s *t*-test for independent samples was used for comparisons between groups. Two-way ANOVA of repeated measures (group and time) was used to evaluate the effect of the program from the baseline to after 12 weeks of intervention. Pairwise comparisons were conducted to explore significant effects, with Bonferroni’s correction applied to adjust for multiple testing. Effect sizes were estimated using the partial eta coefficient squared (η^2^), classifying it as small (η^2^ > 0.01), medium (η^2^ > 0.06), or large (η^2^ > 0.14). The level of significance was set at *p* < 0.05, and the analysis were performed by using SPSS software, version 30.0 (IBM Corp., Armonk, NY, USA).

## 3. Results

### 3.1. Participants 

Table 3 presents the baseline characteristics of the participants at the beginning of the multicomponent program. There were no significant differences between the control and intervention groups in terms of age, body weight, height, or BMI (*p* > 0.05).

### 3.2. Mental Disorders

After 12 weeks of the intervention (Table 4), the teachers in the intervention group showed a significant reduction in symptoms of depression (F(1.24) = 4.950; *p* < 0.05, η^2^ = 0.16), anxiety (F(1.66) = 10.434; *p* < 0.01, η^2^ = 0.29), and stress (F(1.74) = 7.086; *p* < 0.05, η^2^ = 0.21) compared to those in the control group. According to conventional benchmarks, all effects were classified as large.

### 3.3. Biochemical Parameters

The results showed that, after 12 weeks of the multicomponent program, the teachers in the intervention group experienced significant reductions in fasting blood glucose (pre = 104.4 ± 13.9 mg/dL vs. post = 91.7 ± 7.3 mg/dL; F(1.31) = 11.548; *p* < 0.01, η^2^ = 0.27), triglycerides (pre = 128.0 ± 55.4 mg/dL vs. post = 108.7 ± 48.4 mg/dL; F(1.55) = 6.105; *p* < 0.05, η^2^ = 0.17), and VLDL-c (pre = 25.6 ± 11.0 mg/dL vs. post = 21.6 ± 9.7 mg/dL; F(1.24) = 6.731; *p* < 0.05, η^2^ = 0.18) compared to the control group. According to conventional benchmarks, all effects were classified as large.

**Figure 2:**
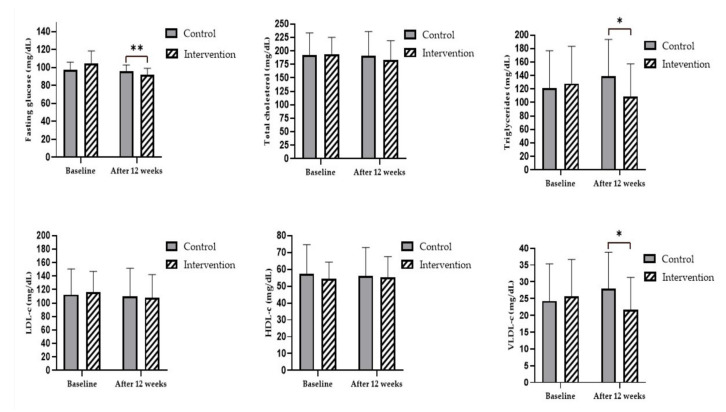
Fasting blood glucose and lipid profile results at baseline and after 12 weeks of multicomponent program. Note. ** *p* < 0.01; * *p* < 0.05.

However, no significant differences were observed in total cholesterol, HDL-c, or LDL-c levels between groups after 12 weeks (*p* > 0.05) (Figure 2).

### 3.4. Immunological Parameters

The results for IgA, IgG, and IgM after 12 weeks of the multicomponent program (Table 5) showed no significant changes in humoral immune response compared to baseline in either group (*p* > 0.05).

## 4. Discussion

The findings of this study demonstrate that a 12-week multicomponent program, consisting of physical exercises, cognitive–behavioral therapy (CBT), and nutritional education, provided significant benefits in terms of the mental and metabolic health of female elementary school teachers with overweight. Consistent reductions in symptoms of depression, anxiety, and stress were observed, as well as improvements in biochemical markers such as fasting glucose, triglycerides and VLDL-c. However, there was no significant change in the humoral immunity. Regarding program adherence, all participants of the intervention group completed the full 12-week intervention. Attendance records indicate 100% participation in the exercise sessions, cognitive–behavioral therapy, and nutritional education, suggesting high feasibility and acceptability of the program.

While previous studies have addressed individual components, this study is among the first to integrate multiple components in a comprehensive approach, analyzing their effects on mental disorders, metabolic health parameters, and immune activity in female elementary school teachers with overweight. The relevance of this study is highlighted by the fact that educators are among the professionals with the highest levels of psychological stress and burnout due to the intense work and emotional demands of the profession [21,22,23].

When teachers are chronically exposed to psychological stress, their susceptibility to a variety of physical and mental health problems may increase. According to Chu et al. [24], chronic stress triggers a response mediated by a complex interaction of nervous, endocrine, and immune mechanisms, activating the hypothalamic–pituitary–adrenal (HPA) axis and the immune system. Activation of the HPA axis leads to the release of corticotropin-releasing hormone (CRH) from the paraventricular nucleus of the hypothalamus into the circulation, stimulating the anterior pituitary gland to secrete adrenocorticotropic hormone (ACTH) into the bloodstream. ACTH then stimulates the adrenal cortex to release the hormone cortisol, which—at high and prolonged levels (hypercortisolemia)—suppresses immune function by inhibiting the production of pro-inflammatory cytokines and reducing the activity of immune cells, particularly lymphocytes. This immunosuppressive effect may increase susceptibility to infections, delay wound healing, and exacerbate inflammatory conditions. Furthermore, hypercortisolemia promotes systemic inflammation through the upregulation of inflammatory mediators, contributing to the pathogenesis of autoimmune diseases, chronic inflammatory disorders, and the degeneration (or wear) of neurons in the hippocampus, thereby increasing susceptibility to depressive disorders [25].

The results of this study demonstrate that the multicomponent program was effective in improving symptoms of depression, anxiety, and stress in female teachers with overweight after 12 weeks of intervention. We identified only one study that combined physical exercise with a form of cognitive–behavioral therapy in teachers. In that study, conducted with elementary school teachers in the northeastern region of the United States, a 6-week virtual intervention was implemented, consisting of 30 min of meditation (mindfulness) followed by 30 min of aerobic exercise, performed twice a week, with each session lasting one hour. The results showed improvements in symptoms of depression, anxiety, and perceived stress at the end of the intervention period [26]. Two systematic reviews analyzing the effectiveness of interventions using cognitive–behavioral therapy and predominantly aerobic physical exercise for mental health in the workplace showed that, in most of the studies reviewed, the interventions were effective in reducing symptoms of depression, anxiety, or both in working populations [27,28].

The mental health benefits observed in teachers in the present study may be related to the synergy between the effects of physical exercise and cognitive–behavioral therapy. Individuals with mental disorders often exhibit reduced expression of brain-derived neurotrophic factor (BDNF), which is essential for synaptic plasticity and neural signal transmission [29]. Physical exercise increases the activity of immune cells and promotes an anti-inflammatory response, which supports neurotransmitter metabolism and neuroendocrine function [30]. Meanwhile, the cognitive–behavioral therapy used in this study focused on the development of socio-emotional and cognitive skills aimed at enhancing teaching effectiveness and reducing emotional and behavioral difficulties. This may have helped modify thoughts and behaviors, enabling participants to face every day professional and personal challenges with greater emotional balance, reduced stress, and improved cognitive clarity [31].

Regarding biochemical parameters significant reductions in fasting blood glucose, triglycerides and VLDL-c levels were observed after 12 weeks in the intervention group. We did not find other studies that implemented three types of interventions within the same program and analyzed their effects on biochemical risk factors for cardiometabolic diseases, making comparisons with other studies difficult. Most existing interventions applied different physical exercise protocols, ketogenic diets, calorie-restriction diets, individualized dietary planning, or a combination of these. These studies have shown that the combination of exercise and dietary interventions leads to better control of glycemic levels and dyslipidemia, particularly after a period of six months [32,33,34,35,36,37,38,39,40].

The lack of research involving more than two intervention strategies highlights the potential of the present study, which, despite its short duration (12 weeks), demonstrated that the synergistic effects of increased physical exercise, reduced stress symptoms, and nutritional education may represent a time-efficient strategy to reduce the risk of developing cardiometabolic diseases in elementary school teachers.

We emphasize that the reduction in fasting blood glucose levels through physical exercise is related to improved insulin sensitivity and increased glucose uptake by muscles [33]. Additionally, stress reduction attenuates the activation of the hypothalamic–pituitary–adrenal (HPA) axis, lowering cortisol levels, and, consequently, decreasing insulin resistance and increasing glucose uptake by both muscles and adipose tissue [41].

With regard to nutritional education activities, teachers may have been encouraged to make healthier food choices, leading to an overall improvement in dietary habits [42]. The observed decreases in blood glucose, triglycerides, and VLDL-c may also have been associated with increased oxidation of blood lipids and free fatty acids (FFAs) by skeletal muscles during physical exercise, which reduces the availability of FFAs to the liver and the formation of new lipids [43]. Furthermore, increased daily energy expenditure—both during and after exercise—as well as improved insulin sensitivity are additional factors that may have contributed to these outcomes [44].

Another possible mechanism is the reduction in stress symptoms, which may have led to decreased activation of the HPA axis and consequently reduced hepatic overproduction of cholesterol-rich lipoproteins, thus lowering VLDL-c levels [41]. Finally, the nutritional education component, which emphasized learning, autonomy, and behavioral change, may have encouraged a reduction in saturated fat consumption among teachers. Additionally, the lack of a structured dietary intake assessment and the absence of a biochemical stress marker (e.g., cortisol) limited our ability to quantify potential biochemical changes that may have contributed to the observed improvements. Therefore, it as not possible to attribute the effects solely to the educational components or to reductions in stress levels.

The lack of significant changes in total cholesterol, LDL-c, and HDL-c levels may indicate that a longer intervention period is necessary to observe such changes or that other factors such as diet and genetics may have influenced these outcomes.

Immunoglobulins are synthesized by plasma cells and provide humoral protection against pathogens in various human biofluids, including blood, saliva, breast milk, cerebrospinal fluid, and gastrointestinal mucus [45]. In the present study, no changes in plasma immunoglobulin levels (IgA, IgG, and IgM) were observed after 12 weeks of the multicomponent program. Systematic reviews have indicated that regular physical exercise may reduce immunoglobulin levels; however, some studies have reported increases, while others have found no association between immunoglobulins and exercise [45,46,47]. These conflicting results across studies may be attributed to differences in study populations and the types, intensities, and durations of the exercise protocols used.

Thus, future studies could investigate whether longer or more intense interventions could generate broader impacts on levels of IgA, IgG, and IgM. It is important to note that this study did not include cellular immune markers, such as cytokines or lymphocyte subsets, due to resource limitations. Future research should include both humoral and cellular components to better understand the immune response.

Despite the interesting findings, this study has several limitations. The small sample size and inclusion of only female teachers in both the control and intervention groups limit the generalizability of the results, particularly with respect to causal inferences. These factors also raise concerns about internal validity, which are inherent to quasi-experimental designs. Notably, the inclusion of only female teachers is supported by demographic data from the 2019 Brazilian Basic Education Census, which indicate that women comprise over 80% of the teaching workforce in Brazil.

Additionally, no nutritional assessment was conducted to analyze dietary changes resulting from the nutritional education activities proposed in the program, which limits the evaluation of the intervention’s overall effectiveness.

However, the results are still significant, considering that this study was conducted between September and November—the final quarter of the school year for teachers—which is typically a period of overload due to accumulated responsibilities. It is worth noting that this study represents an advance in research on teacher health, suggesting that a short-term program aimed at increasing physical activity, enhancing stress management, and promoting learning, autonomy, and behavior change through nutritional education can be a time-efficient strategy for improving the physical and mental health of basic education teachers.

Furthermore, this program may serve as a foundation for proposing a public health policy for teachers in basic education across different educational contexts. Nevertheless, the relatively short duration of the intervention may restrict the generalizability of the results. Therefore, further studies with experimental designs, larger samples, and longer intervention periods are needed to confirm the beneficial effects of multicomponent programs—those with more than two intervention elements—on physical and mental health outcomes in basic education teachers.

## 5. Conclusions

In summary, the 12-week multicomponent program implemented in this study was effective in improving mental health and reducing cardiometabolic risk factors in female teachers with overweight. However, it did not lead to improvements in humoral immune response. These results reinforce the importance of integrated interventions to promote health and well-being in high occupational stress conditions, suggesting that this program may be a simple and viable strategy to prevent disease and improve the quality of life of education professionals.

Implementing similar initiatives in school settings can not only reduce absenteeism and time-off costs but also contribute to a healthier, more productive, and welcoming educational environment, benefiting both staff and students.

## Figures and Tables

**Table 1:** Exercise program.

Warm-Up		
Exercise		Repetition
Shoulder and hip flexion posture with spinal extension and posterior chain activation. 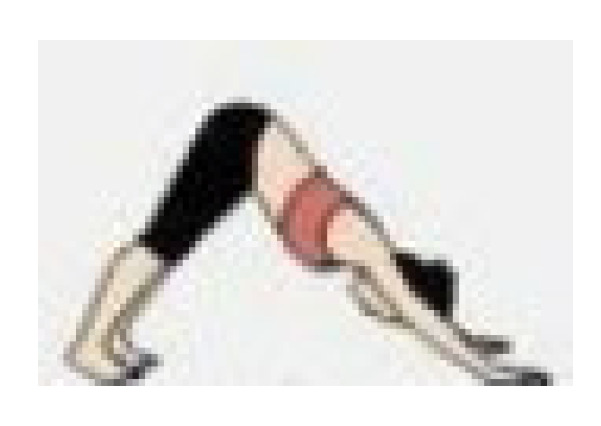		5 repetitions
Knee and hip flexion and extension. 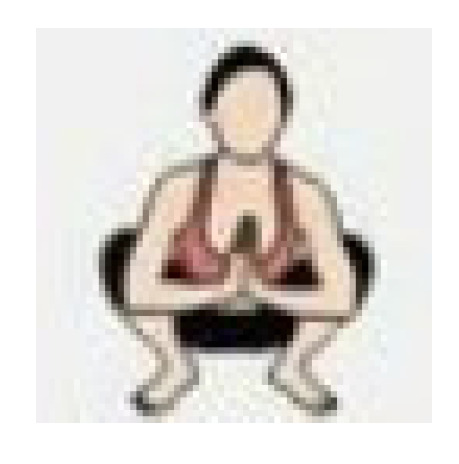		5 repetitions
Hip and knee flexion and extension in a plank position. 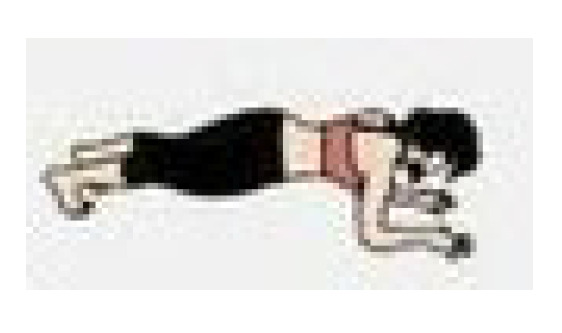		5 repetitions
Dynamic mobility stretching for hips and shoulders (lunge with arms raised). 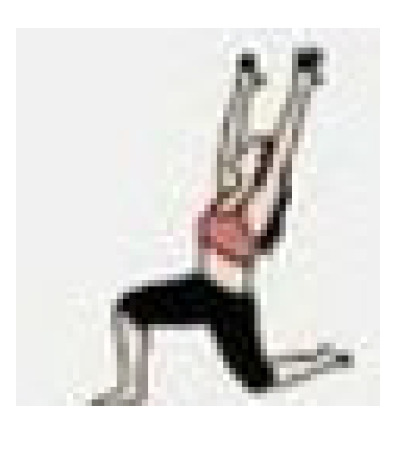		5 repetitions per leg
Bird dog. 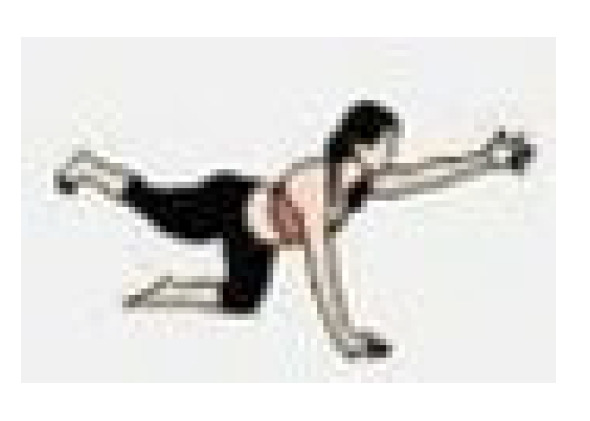		5 repetitions per arm
Lumbar flexion. 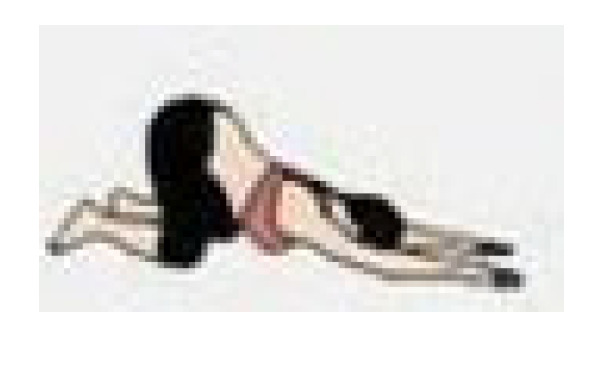		5 repetitions
Cat–cow stretch. 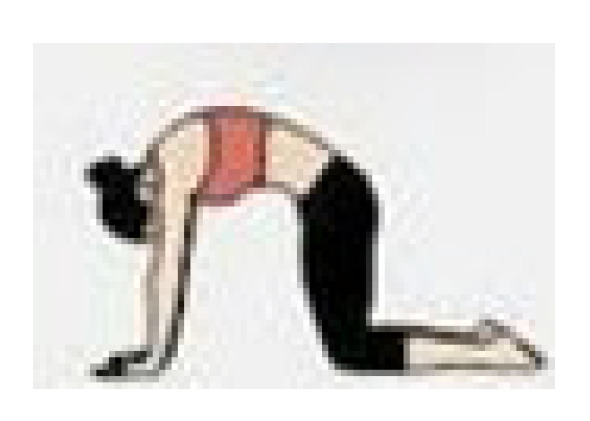		5 repetitions
Twisting lunge or reverse lunge pose. 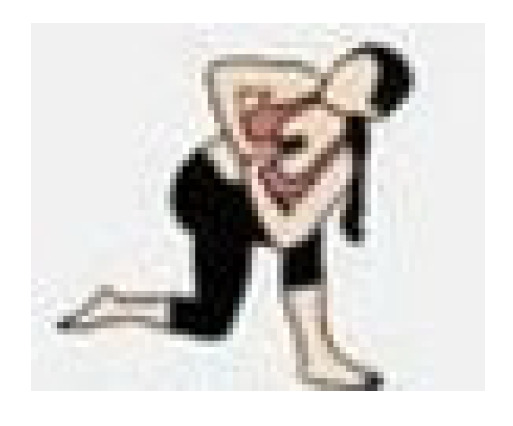		5 repetitions per side
Lateral trunk stretch with floor support (gate pose). 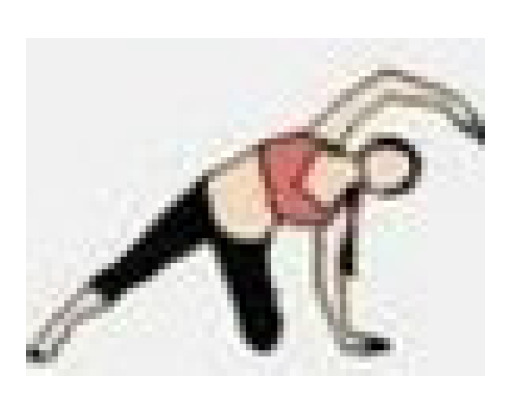		5 repetitions per side
Hip abduction. 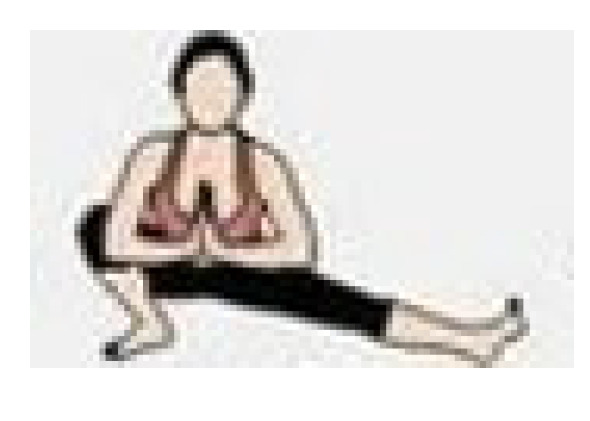		5 repetitions per leg
Main part		
Weeks	Muscle/exercise	Sets/repetition
1–4	Chest: dumb-bell fly (flat) Back: lat pulldown (front and behind the neck) Shoulders: lateral raise Biceps: barbell curl (dumbbells) Triceps: triceps pushdown Quadriceps: squat Hamstrings: stiff Glutes: hip thrust Calves: standing calf raise Rectus abdominis: Swiss ball crunch Treadmill (30 s) Stationary bike (30 s)	3 × 15
5–8		3 × 12
9–12		3 × 10
Return to calm		
Main muscle groups	Execution	Duration
Hamstring stretch	Lie on your back and extend one leg upward, holding it behind the thigh or calf. Keep the other leg bent or flat on the floor.	20 to 30 s
Quadricep stretch	Stand up, grasp one ankle with the corresponding hand, pulling it toward your buttocks. Keep your knees aligned and your torso upleft.	20 to 30 s
Gluteal stretch	Lie on your back, cross one leg over the other, placing the ankle on the opposite knee. Pull the lower leg toward your chest.	20 to 30 s
Chest Stretch	Stand with your arms extended behind you, interlocking your fingers. Gently lift your arms upward, opening your chest.	20 to 30 s
Back stretch	Sit or stand, interlock your fingers and extend your arms forward, rounding your back and pushing your hands away from your body.	20 to 30 s
Neck stretch	Tilt your head to one side, bringing your ear toward your shoulder, and gently press with the opposite hand	20 to 30 s

**Table 2:** Topics covered in cognitive–behavioral therapy interventions.

Module	Contents	Objectives	Duration
1 Social skills training and emotional regulation mental health education and cognitive strategies Solution-focused therapy and emotional expression	Discussion groups Psychoeducation Teachers’ suggestion box	Promote cognitive restructuring, awareness and encourage cognitive restructuring and autonomy	1 h
2 Problem solving and SMART goal setting Guided imagery and cognitive rehearsal	Problem identification and goal setting Vision of the future	Reduce feelings of helplessness and increase the perception of control over professional reality	1 h
3 Cognitive restructuring and positive self-image Beck’s cognitive triad model	Mirror dynamics Cognitive triad, thought assessment, refocusing Mirror dynamics	Reduce discomfort regarding self-image and professional perception	1 h

Note: SMART—specific, measurable, attainable, relevant, and timely.

**Table 3:** The participants’ characteristics.

Variables	Control (*n* = 16)	Intervention (*n* = 17)	
	Mean ± SD	Mean ± SD	*p*-Value
Age (years)	43.1 ± 6.5	43.8 ± 7.7	0.763
Body weight (kg)	83.2 ± 9.1	80.9 ± 8.7	0.470
Height (m)	1.62 ± 0.0	1.61 ± 0.0	0.776
BMI (kg/m^2^)	31.8 ± 3.9	31.0 ± 3.2	0.577

Note: Body mass index (BMI), meters (m).

**Table 4:** Results of depression, anxiety, and stress symptoms at baseline and after 12 weeks of the multicomponent program.

Symptoms	Control (*n* = 16)		Intervention (*n* = 17)	
	Baseline	After 12 Weeks	Baseline	After 12 Weeks
	Mean ± SD	Mean ± SD	Mean ± SD	Mean ± SD
Depression (Score)	3.2 ± 3.2	2.4 ± 3.2	5.6 ± 5.8	2.1 ± 2.9 *
Anxiety (Score)	3.1 ± 2.7	3.8 ± 4.2	6.5 ± 5.1	2.7 ± 3.2 **
Stress (Score)	5.2 ± 2.1	5.4 ± 4.9	8.7 ± 6.3	4.2 ± 4.4 *

Note: * *p* < 0.05; ** *p* < 0.01.

**Table 5:** Immunoglobulin results at the beginning and after 12 weeks of multicomponent program.

Immunoglobulins	Control (*n* = 16)		Intervention (*n* = 17)	
	Baseline	After 12 Weeks	Baseline	After 12 Weeks
	Mean ± SD	Mean ± SD	Mean ± SD	Mean ± SD
IgA (mg/dL)	225.3 ± 73.9	224.4 ± 73.9	232.6 ± 87.3	227.1 ± 75.9
IgG (mg/dL)	1.152 ± 185.8	1.158 ± 205.7	1.138 ± 271.2	1.180 ± 287.5
IgM (mg/dL)	102.1 ± 41.9	100.2 ± 32.7	100.0 ± 48.6	99.4 ± 49.0

## Data Availability

The raw data supporting the conclusions of this article will be made available by the authors on request due to being part of a larger study whose data are still being analyzed for other manuscripts’ publication.
